# A pretreatment multiphasic CT-based decision-support model for differentiating pediatric hepatoblastoma from focal nodular hyperplasia

**DOI:** 10.3389/fonc.2026.1923907

**Published:** 2026-08-24

**Authors:** Lizhu Cai, Yun Peng

**Affiliations:** Department of Radiology, MOE Key Laboratory of Major Diseases in Children, Beijing Children’s Hospital, Capital Medical University, National Center for Children’s Health, Beijing, China

**Keywords:** deep learning, focal nodular hyperplasia, hepatoblastoma, multiphasic contrast-enhanced CT, quantitative enhancement features

## Abstract

**Background:**

Hepatoblastoma (HB) and focal nodular hyperplasia (FNH) require markedly different management strategies in children, but differentiation can be challenging when multiphasic computed tomography (CT) findings are atypical or overlapping. This study aimed to develop and temporally validate a pretreatment multiphasic CT-based fusion model combining deep learning (DL) predictions with quantitative enhancement features for pediatric HB–FNH differentiation and to evaluate the potential value of model assistance for junior-radiologist interpretation.

**Methods:**

This retrospective study included 612 children who underwent pretreatment multiphasic CT between January 2011 and September 2025, including 523 with HB and 89 with FNH. A chronological split on July 1, 2023 assigned 491 patients to model development and 121 patients to temporal testing. Three phase-specific DL models based on 2.5D ResNet-18 were trained using precontrast, arterial-phase, and portal venous-phase images, and their predicted probabilities were averaged to generate DL-3Phase. Quantitative enhancement features from standardized manual region-of-interest attenuation measurements were used to train a regularized logistic regression model, termed the quantitative enhancement feature (QEF) model. DL+QEF was prespecified as the unweighted average of the DL-3Phase and QEF model probabilities. Performance was assessed using the area under the receiver operating characteristic curve (AUC), diagnostic accuracy metrics, calibration, decision curve analysis, and comparison with radiologist interpretation.

**Results:**

In the temporal test cohort, DL+QEF achieved an AUC of 0.980, an accuracy of 94.2%, an HB sensitivity of 94.9%, and an FNH specificity of 92.9%. DL+QEF showed better discrimination than DL-3Phase and the best-performing single-phase DL model, and combined the high HB sensitivity of DL-3Phase with the high FNH specificity of the QEF model. In the pathology-confirmed test subset, the AUC was 0.972 and accuracy was 92.9%. Junior-radiologist accuracy was 88.4% during unaided reading and 95.0% during model-assisted reading (exact McNemar *p =* 0.0078). In reader-defined atypical cases, DL+QEF had higher accuracy than the junior radiologist (90.2% vs. 68.3%).

**Conclusion:**

The pretreatment multiphasic CT-based DL+QEF model showed favorable diagnostic performance for pediatric HB–FNH differentiation and may serve as an adjunct to CT interpretation, with potential utility in reader-defined atypical or diagnostically uncertain cases.

## 1 Introduction

Hepatoblastoma (HB) is the most common primary malignant liver tumor in children. Complete surgical resection remains a cornerstone of treatment and is often preceded by neoadjuvant chemotherapy to improve resectability (1, 2). By contrast, focal nodular hyperplasia (FNH) is a benign pediatric hepatic lesion that is generally managed conservatively (3, 4), whereas surgery is reserved mainly for symptomatic lesions or persistent diagnostic uncertainty (4–6). Given their fundamentally different biological behavior, management pathways, and prognostic implications, reliable pretreatment differentiation between HB and FNH is clinically consequential. Accurate distinction may help avoid unnecessary biopsy or surgery for FNH (1, 7), while reducing the risk of misclassifying HB as a benign lesion, which could delay systemic treatment or timely surgical planning. However, HB and FNH may show overlapping enhancement patterns on multiphasic CT, particularly when their imaging appearances are atypical (8). Although serum AFP is routinely incorporated into the diagnostic workup, physiological elevation during infancy and occasional AFP elevation in patients with FNH limit its specificity (9–14).

In adults, FNH typically shows marked arterial-phase enhancement, becomes isoattenuating to the liver on portal venous or delayed phases, and may show a central scar with delayed enhancement (15, 16). In children, however, FNH is uncommon and may show more variable imaging appearances (17). Compared with the classic adult imaging pattern, pediatric FNH may present with larger lesion size or multifocal disease, and a visible central scar is not consistently present. In one pediatric imaging series, a central scar was reported in approximately 20% of lesions (17, 18). Prior oncologic treatment may further complicate image interpretation and contribute to diagnostic uncertainty (19). Similarly, although HB often appears as a large heterogeneous mass with necrosis, hemorrhage, or calcification, some lesions may show relatively homogeneous enhancement and well-defined margins, thereby overlapping with benign hypervascular lesions (18, 20, 21). The main diagnostic challenge therefore lies not in typical HB or typical FNH, but in the smaller subset of cases in which enhancement behavior or lesion morphology deviates from typical imaging patterns. In such cases, visual interpretation may become more dependent on reader experience. In addition, the enhancement characteristics of pediatric HB and FNH differ from those of adult hepatocellular carcinoma and other common adult focal liver lesions (22). Therefore, diagnostic frameworks developed for adult liver lesion characterization should not be directly extrapolated to pediatric HB–FNH differentiation (19, 23).

MRI, particularly hepatobiliary phase imaging, provides important diagnostic information for FNH (1, 24, 25). In routine practice, however, complete MRI examinations are not available for all children. In historical datasets and pretreatment settings requiring timely assessment, multiphasic contrast-enhanced CT often serves as the initial or primary imaging examination (22). These circumstances underscore the need to determine whether pretreatment CT information can be used more effectively to support HB–FNH differentiation.

Multiphasic contrast-enhanced CT provides information on precontrast lesion attenuation, arterial- and portal venous-phase enhancement, temporal changes in enhancement, and intratumoral heterogeneity. Conventional interpretation relies mainly on radiologists’ assessment of multiphasic enhancement patterns. This process is partly experience-dependent and may become less consistent across readers when lesions deviate from typical imaging patterns. Quantitative enhancement parameters, including arterial-phase enhancement, tumor-to-liver attenuation ratios, and attenuation changes across phases, can translate visual impressions into structured, reproducible measurements (26). However, manual attenuation measurements alone cannot fully capture intratumoral spatial heterogeneity. Deep learning can learn image features directly from multiphasic CT images, including features that are not easily captured by predefined quantitative measurements (27, 28). However, deep learning models do not typically encode attenuation-based enhancement kinetics in a readily interpretable form. Combining multiphasic CT-based deep learning with quantitative enhancement features may therefore integrate spatial image information with interpretable attenuation-based enhancement-kinetic assessment. This combined approach may provide objective support for pediatric HB–FNH cases in which conventional visual interpretation remains uncertain.

To our knowledge, no previous study has specifically developed a pretreatment multiphasic CT-based AI model to differentiate pediatric HB from FNH. In this study, we aimed to develop and temporally validate a CT-only fusion model that combines multiphasic deep learning predictions with quantitative enhancement features for pretreatment differentiation between pediatric HB and FNH. We also compared model performance with that of radiologists with different levels of experience and evaluated junior-radiologist performance during unaided and model-assisted reading. As an exploratory analysis, we evaluated cases initially categorized by the junior radiologist as atypical or diagnostically uncertain on CT. We further assessed the robustness and potential clinical applicability of the model in the pathology-confirmed subset, across different enhancement phenotypes, and with enhancement features derived from whole-tumor mean attenuation.

## 2 Materials and methods

### 2.1 Study design and patients

**Figure 1:**
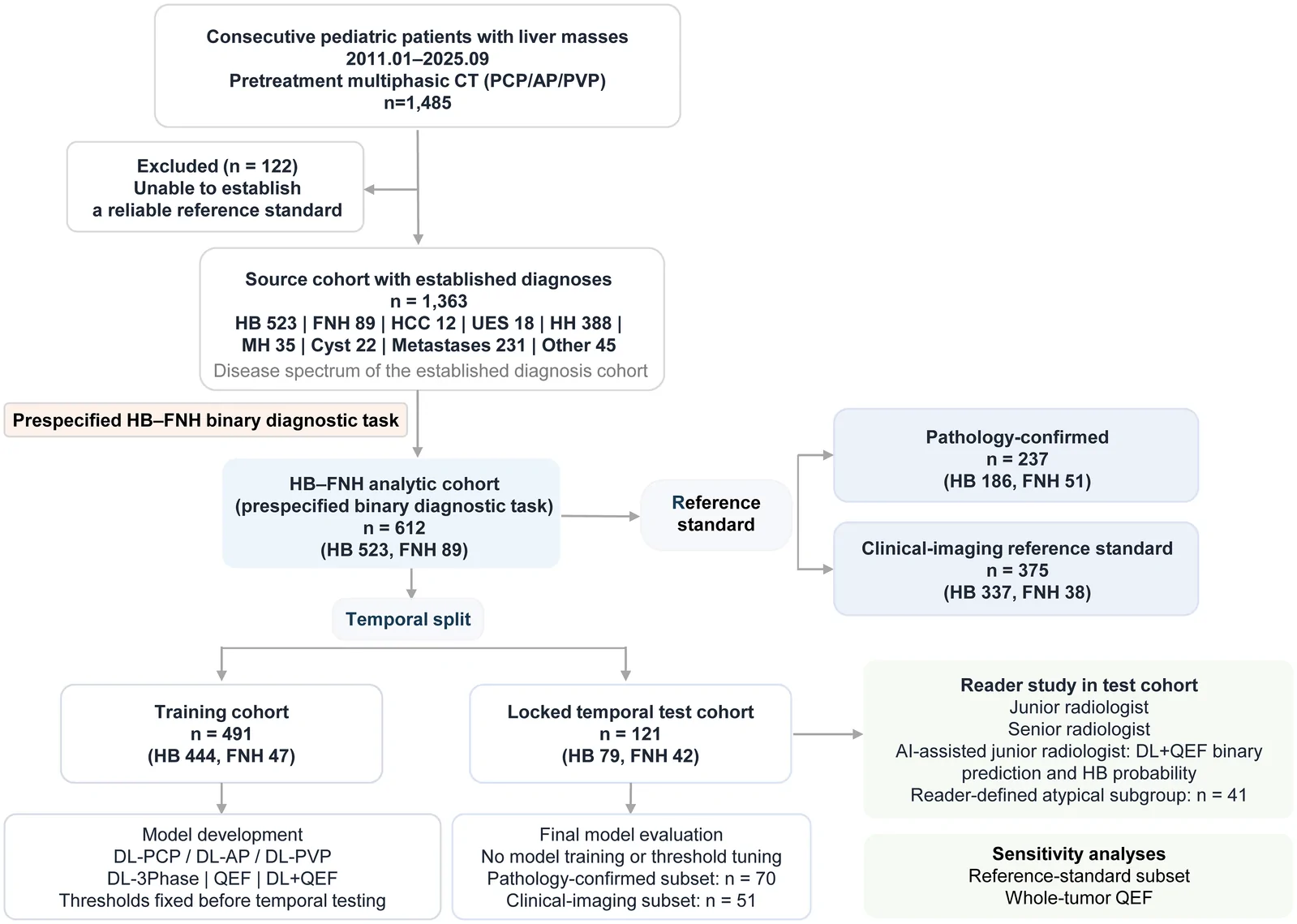
Study flowchart and analysis cohorts. The study was derived from a consecutive cohort of 1,485 pediatric patients with liver masses who underwent pretreatment multiphasic CT. After exclusion of 122 patients without a reliable reference standard, 1,363 patients with established diagnoses were identified, from which the prespecified HB–FNH analytic cohort (HB, n = 523; FNH, n = 89) was selected. Patients were split temporally at July 1, 2023 into a training cohort for model development and a locked temporal test cohort for final evaluation. Reader study and sensitivity analyses are summarized for the corresponding cohorts. HB, hepatoblastoma; FNH, focal nodular hyperplasia; HCC, hepatocellular carcinoma; UES, undifferentiated embryonal sarcoma; HH, hepatic hemangioma; MH, mesenchymal hamartoma; PCP, precontrast phase; AP, arterial phase; PVP, portal venous phase; QEF, quantitative enhancement feature model.

This single-center retrospective diagnostic modeling study screened consecutive pediatric patients who underwent pretreatment multiphasic contrast-enhanced CT for a suspected liver mass at Beijing Children’s Hospital, Capital Medical University, between January 2011 and September 2025. The screening and exclusion process is summarized in **Figure 1**. Of 1,485 patients screened, 122 were excluded because a reliable reference-standard diagnosis could not be established from the available pathological, clinical, or follow-up information. Among the remaining 1,363 patients with an established diagnosis, the study prespecified a binary diagnostic analysis of hepatoblastoma (HB) versus focal nodular hyperplasia (FNH).

Eligible patients were younger than 18 years, had a final diagnosis of HB or FNH, underwent CT before initial treatment, and had complete precontrast, arterial-phase, and portal venous-phase images available for analysis. Lesions outside the prespecified HB–FNH diagnostic task were not included. When multiple pretreatment CT examinations were available for the same patient, only one examination was retained.

A total of 612 patients met the eligibility criteria and were included in the final analysis. Patients examined before July 1, 2023, were assigned to the training cohort, and those examined on or after July 1, 2023, were assigned to the temporal test cohort (**Figure 1**). The model was developed in the training cohort and evaluated only in the temporal test cohort, which was not used during model development.

This study was approved by the Ethics Committee of Beijing Children’s Hospital, Capital Medical University ([2026]-E-012-R). The requirement for written informed consent was waived because of the retrospective design and the use of de-identified data. The study was reported in accordance with the Standards for Reporting Diagnostic Accuracy Studies (STARD).

### 2.2 Reference standard

A predefined composite reference standard was used. Histopathological confirmation served as the primary reference standard whenever tissue diagnosis was available and was obtained in 237 patients (HB, n = 186; FNH, n = 51). For the remaining 375 patients (HB, n = 337; FNH, n = 38), the final diagnosis was based on multidisciplinary assessment documented in the medical records and/or longitudinal clinical and imaging follow-up, without access to the study model outputs. One radiologist retrospectively extracted the clinical-imaging reference-standard information from the electronic medical records according to prespecified criteria. The reference diagnosis was based on the final documented clinical assessment together with the available longitudinal clinical and imaging evidence and was not newly assigned using study-specific QEF measurements or model outputs.

For FNH, the diagnosis was based on characteristic imaging findings together with clinical presentation and serum alpha-fetoprotein (AFP) level and was supported by either lesion stability without evidence of malignant behavior during at least 24 months of follow-up or documented multidisciplinary consensus.

For HB, the diagnosis was based on characteristic malignant imaging findings, clinical presentation, and serum AFP level, supported by a documented multidisciplinary diagnosis, disease progression during follow-up, or response to chemotherapy with tumor regression and a reduction in AFP.

To evaluate the potential influence of reference standard heterogeneity, prespecified sensitivity analyses were performed separately in the pathologically confirmed subset and in the clinical-imaging reference standard subset.

### 2.3 CT acquisition and tumor segmentation

All patients underwent pretreatment abdominal multiphasic contrast-enhanced CT. CT examinations were performed using a GE Discovery CT750 HD or GE Revolution CT scanner (GE Healthcare, Milwaukee, WI, USA). Dual-energy CT was acquired using rapid kVp switching between 80 and 120 kVp. Tube current was adjusted according to body weight (200, 240, 280, and 320 mA for body weights of 0–15.9 kg, 16–31.9 kg, 32–39.9 kg, and 40–50 kg, respectively). Images were acquired with a detector width of 40 mm and a gantry rotation time of 0.5 s.

Precontrast, arterial-phase, and portal venous-phase images were available for all patients. Iohexol (350 mg I/mL) was administered intravenously using a power injector at a dose of 1.6 mL/kg (0–15.9 kg) or 1.4 mL/kg (16–50 kg), with an injection duration of 17–21 seconds. Arterial-phase and portal venous-phase images were acquired approximately 21 and 55 seconds, respectively, after the start of contrast injection. Images were reconstructed at a section thickness of 5 mm with 50% adaptive statistical iterative reconstruction-V (ASIR-V). Patient-level CTDIvol and DLP records were not consistently retrievable throughout the extended retrospective study period and therefore could not be summarized for the full cohort.

CT examinations were performed without sedation when adequate cooperation could be achieved. Children unable to cooperate underwent oral sedation with 10% chloral hydrate (0.4 mL/kg) according to institutional pediatric imaging practice.

For each patient, lesions were manually delineated on CT images from each phase using 3D Slicer (version 5.8.1). Whole-tumor volumes of interest (VOIs) were delineated by two radiologists with 4 and 6 years of experience, each of whom annotated a separate subset of cases. All VOIs were subsequently reviewed and corrected, when necessary, by a senior pediatric radiologist with 21 years of experience. Segmentation was performed without access to the reference-standard diagnosis or model outputs. In patients with multifocal disease, all visible lesions were included in a single patient-level mask, and each patient generated one model prediction. The VOIs were used for tumor localization, tumor-centered patch extraction for DL model development, and calculation of whole-tumor mean CT attenuation values in the whole-tumor quantitative enhancement sensitivity analysis.

### 2.4 Quantitative enhancement features

Manual small-ROI attenuation measurements were used to derive the inputs for the quantitative enhancement feature model (QEF). Two radiologists independently performed phase-specific attenuation measurements using the same predefined ROI placement rules. For each reader and each CT phase, two tumor ROIs of approximately 2 cm² were placed in representative solid enhancing regions while avoiding necrosis, cystic degeneration, hemorrhage, calcification, vessels, and obvious artifacts. The two measurements were averaged to obtain a reader-specific mean attenuation value, and the reader-specific means from the two radiologists were subsequently averaged to obtain the final phase-specific tumor attenuation value used for QEF calculation. In patients with multifocal disease, measurements were obtained from the largest or most representative lesion. A liver parenchymal ROI of approximately 2 cm² was placed in homogeneous uninvolved liver parenchyma, avoiding visible vessels and bile ducts. An abdominal aortic ROI of approximately 0.2 cm² was placed at the level of the celiac trunk, and a portal venous ROI of approximately 0.2 cm² was placed on portal venous-phase images. Quantitative enhancement features representing enhancement magnitude, enhancement changes across phases, tumor-to-liver attenuation differences and ratios, vessel-normalized attenuation ratios, and washout-related indices were constructed from precontrast, arterial-phase, and portal venous-phase attenuation measurements (**Supplementary Methods S1**).

Interobserver agreement was assessed using the reader-specific mean attenuation values obtained by the two radiologists. Intraclass correlation coefficients were calculated using a two-way random-effects model for absolute agreement and single measurements [ICC(2,1)].

### 2.5 Model development

Model inputs were limited to pretreatment multiphasic CT images and CT-derived quantitative enhancement features. Age, sex, and laboratory variables were not included as model inputs. Age and sex were used only for baseline characterization and subgroup analyses, and laboratory variables were used only as supporting information for the clinical-imaging reference standard. This design was intended to evaluate the diagnostic value of multiphasic CT information for differentiating HB from FNH, including image patch-based deep learning features and CT attenuation-based quantitative enhancement features, while reducing the possibility that model performance would be driven mainly by clinical factors such as age or AFP.

**Figure 2:**
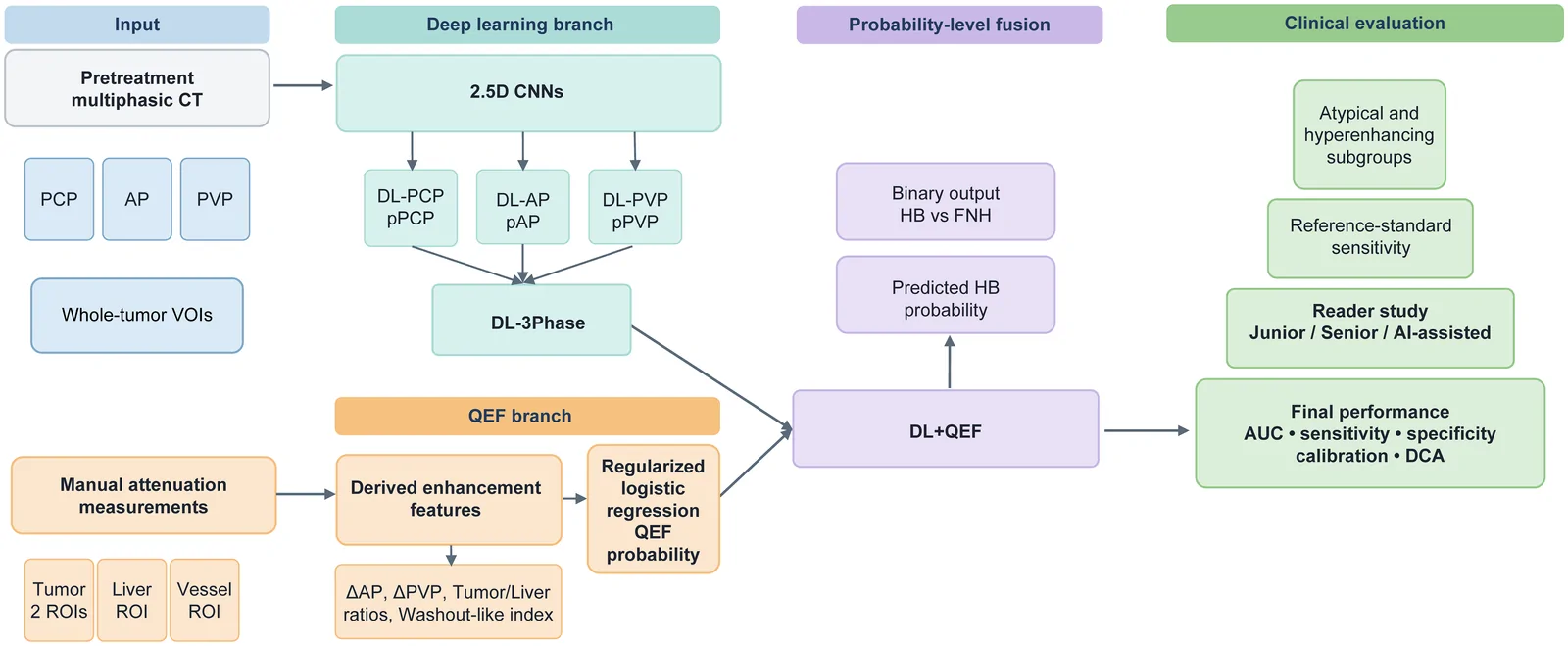
Multiphasic CT deep learning and quantitative enhancement feature model workflow. Pretreatment precontrast, arterial-phase, and portal venous-phase CT images were used to develop separate single-phase DL models. The pPCP, pAP, and pPVP denote the predicted probabilities of HB from the corresponding phase-specific DL models. Quantitative enhancement features were derived from standardized attenuation measurements and used to construct the QEF model. DL-3Phase was obtained by probability averaging of the three single-phase DL models, and DL+QEF was obtained by probability averaging of DL-3Phase and QEF. DL, deep learning; QEF, quantitative enhancement feature model; PCP, precontrast phase; AP, arterial phase; PVP, portal venous phase; VOI, volume of interest; CNN, convolutional neural network; DCA, decision curve analysis.

The overall workflow for multiphasic CT-based DL model development, QEF construction, and DL+QEF fusion is shown in **Figure 2**. Separate single-phase DL models based on a 2.5D ResNet-18 architecture were developed for the precontrast, arterial, and portal venous phases and were denoted as DL-PCP, DL-AP, and DL-PVP, respectively. For DL preprocessing, CT images and corresponding masks were resampled to an isotropic voxel spacing of 1.0 × 1.0 × 1.0 mm³ using B-spline interpolation for CT images and nearest-neighbor interpolation for masks. No interphase image registration was performed because the three phase-specific models were developed independently using the corresponding phase-specific images and segmentations, and multiphasic information was integrated at the probability level. The resampled whole-tumor VOIs were used to localize each lesion and extract fixed-size tumor-centered patches from the corresponding CT phase. Each patch measured 16 × 128 × 128 voxels, with 16 consecutive axial slices used as input channels. Where a fixed-size patch extended beyond the image boundaries, CT image values were padded with −1024 HU and mask values were padded with 0. CT attenuation values were subsequently clipped to −200 to 300 HU and standardized using the mean and standard deviation of each patch. The models were initialized without pretrained weights. The first convolutional layer was modified to accept 16 input channels, and the final fully connected layer was replaced with a single-output layer. During training, data augmentation consisted of random displacement of the patch center by up to two slices in the axial direction and eight pixels in each in-plane direction. Models were optimized using binary cross-entropy with logits and the AdamW optimizer, with a learning rate of 3 × 10^-4^, a weight decay of 1 × 10^-4^, a batch size of 8, and 10 training epochs. A fixed learning rate was used without a learning-rate scheduler or early stopping. The random seed was set to 2026, and no formal grid search, random search, or automated hyperparameter optimization was performed for the DL branch. Stratified five-fold cross-validation was performed within the training cohort. Class imbalance was addressed using inverse-frequency weighted sampling, and the checkpoint with the highest validation AUC was retained within each fold. Out-of-fold probabilities were generated for patients in the training cohort using the model from the fold in which each patient served as a validation case. For the temporal test cohort, each case was evaluated using all five fold-specific models, and the five predicted probabilities were averaged to obtain the final probability for each single-phase DL model. The temporal test cohort was not used for model fitting, model selection, hyperparameter determination, or threshold selection; all deterministic preprocessing steps were applied identically to the training and temporal test cohorts.

The QEF model was constructed using class-weighted L2-regularized logistic regression. Missing feature values were imputed using the median, and all features were standardized using z-score transformation. A nested cross-validation procedure was used to generate training-cohort out-of-fold probabilities. Within each outer cross-validation fold, the imputation and standardization parameters were estimated using only the corresponding outer training partition. The regularization parameter was selected through inner cross-validation within that partition, without access to the outer held-out fold. The fitted preprocessing pipeline and QEF model were then applied to the outer held-out fold to generate out-of-fold probabilities. After all out-of-fold predictions had been generated, the complete QEF pipeline was refitted using the full 491-patient training cohort, with the regularization parameter selected by cross-validation within the full training cohort, and was then applied unchanged to the temporal test cohort. The definitions and units of all QEFs, together with the final model coefficients, imputation values, and standardization parameters, are provided in **Supplementary Methods S1**.

The primary fusion strategy was prespecified as unweighted probability averaging. DL-3Phase was defined as the mean predicted probability of HB from the three single-phase models. DL+QEF was defined as the mean predicted probability of HB from DL-3Phase and the QEF model. A fixed probability threshold of 0.50 was used for the single-phase DL models, DL-3Phase, QEF, and DL+QEF. No classification threshold was optimized using the temporal test cohort. This approach introduced no additional trainable parameters and was used to reduce the risk of overfitting associated with a second-level fusion model, given the limited sample size and class imbalance. As a sensitivity analysis, we also fitted a logistic regression late-fusion model using out-of-fold predicted probabilities from the training cohort and applied the fitted model to the temporal test cohort.

Model development and image processing were performed using Python 3.10, PyTorch 2.5.1 with CUDA 12.1, torchvision 0.20.1, SimpleITK 2.5.3, scikit-learn 1.7.2, and NumPy 1.26.4 on a workstation running Ubuntu 22.04 LTS and equipped with an NVIDIA Tesla V100-SXM2 GPU.

### 2.6 Reader study

Two radiologists with 4 and 21 years of experience participated in the reader study conducted in the temporal test cohort. Images were reviewed using RadiAnt DICOM Viewer (version 2025.1, 64-bit). Readers were permitted to scroll through the complete image series, adjust window width and level, zoom, and pan, and use multiplanar reformations as needed. During the initial unaided session, both radiologists independently classified each case as HB or FNH using pretreatment precontrast, arterial-phase, and portal venous-phase CT images. Cases were presented in randomized order, and both radiologists were blinded to the reference-standard diagnosis, clinical and laboratory information, and model outputs. During this session, the junior radiologist also recorded whether each case had an atypical imaging appearance or caused diagnostic uncertainty. Cases meeting either criterion were categorized as reader-defined atypical cases. Some radiologists had overlapping roles across the reader study, tumor segmentation, QEF measurement, and reference-standard data extraction. The initial unaided reading session was completed before any subsequent tumor segmentation, QEF measurement, reference-standard data extraction, or review of model outputs.

After a 4-week washout period, the junior radiologist independently reassessed all cases in a newly randomized order with access to the binary classification generated by DL+QEF and the predicted probability of HB. The unaided diagnosis was retained, and a separate assisted diagnosis was recorded during the second session.

### 2.7 Subgroup, sensitivity, and exploratory analyses

Model performance was evaluated in subsets defined by the reference standard, age (<5 years vs. ≥5 years), sex, and cases categorized by the junior radiologist as atypical or diagnostically uncertain. Exploratory scanner analyses assessed the association between scanner type and diagnosis within each cohort and evaluated scanner-stratified DL+QEF performance in the temporal test cohort. As an exploratory analysis, a composite quantitative hyperenhancement phenotype was defined using five indices derived from the manual small-ROI attenuation measurements: aorta-normalized arterial-phase enhancement, absolute arterial-to-precontrast enhancement, liver-normalized arterial-phase enhancement, the arterial-phase tumor-to-liver attenuation ratio, and the portal venous-phase tumor-to-liver attenuation ratio. For each index, the threshold was defined as the 75th percentile of its distribution in the training cohort, and a lesion was classified as hyperenhancing when at least one of the five indices exceeded its corresponding threshold. A stricter definition of marked hyperenhancement was additionally evaluated using the 90th-percentile thresholds for the same five indices.

To assess whether the measurement strategy influenced model performance, the same categories of quantitative enhancement features were constructed using whole-tumor mean CT attenuation values derived from the whole-tumor VOIs. A whole-tumor QEF model (WT-QEF) and the corresponding DL+WT-QEF fusion model were then developed and compared with the primary QEF and DL+QEF models. Finally, as a threshold sensitivity analysis, model-specific classification thresholds were selected by maximizing the Youden index using training-cohort out-of-fold predictions and were applied without modification to the temporal test cohort.

### 2.8 Statistical analysis

Continuous variables were presented as medians with interquartile ranges and were compared between groups using the Mann-Whitney U test. Categorical variables were presented as counts and percentages and were compared using the chi-square test or Fisher’s exact test, as appropriate.

Model performance was assessed using the area under the receiver operating characteristic curve (AUC), accuracy, sensitivity, specificity, balanced accuracy, positive predictive value, and negative predictive value. HB was defined as the positive class and FNH as the negative class. Accordingly, sensitivity represented the sensitivity for HB, whereas specificity represented the specificity for FNH. The 95% confidence intervals for AUC and balanced accuracy were estimated using 5,000 bootstrap resamples stratified by diagnosis, whereas Wilson score intervals were calculated for accuracy, sensitivity, specificity, positive predictive value, and negative predictive value. Paired differences in AUC and accuracy were estimated with 95% confidence intervals using 5,000 diagnosis-stratified paired bootstrap resamples. AUCs were compared between models using the DeLong test. Paired classification results were compared using the exact McNemar test.

Model calibration was evaluated using the Brier score, calibration intercept, calibration slope, expected calibration error, and calibration curves. Calibration curves were constructed using five equal-width probability intervals, whereas expected calibration error was calculated using ten equal-width probability intervals. Clinical net benefit was assessed using decision curve analysis. The prespecified primary comparison was the AUC of DL+QEF versus DL-3Phase in the temporal test cohort. Comparisons with the single-phase DL models, QEF, and radiologists, together with subgroup and sensitivity analyses, were considered secondary or exploratory. All statistical tests were two-sided. A two-sided *p* < 0.05 was considered statistically significant for prespecified primary comparisons, whereas p values from secondary and exploratory analyses were interpreted descriptively without adjustment for multiplicity.

## 3 Results

### 3.1 Patient characteristics

A total of 612 pediatric patients with liver tumors were included in the final analytic cohort, including 523 with hepatoblastoma (HB) and 89 with focal nodular hyperplasia (FNH). According to the temporal split, 491 patients were assigned to the training cohort, including 444 with HB and 47 with FNH, and 121 were assigned to the temporally independent test cohort, including 79 with HB and 42 with FNH. The proportion of FNH was higher in the temporal test cohort than in the training cohort (34.7% vs. 9.6%, *p* < 0.001).

Overall, 237 patients had pathologically confirmed diagnoses, including 186 with HB and 51 with FNH. The remaining 375 patients were diagnosed using a clinical-imaging reference standard, including 337 with HB and 38 with FNH. In the temporal test cohort, 70 patients had pathologically confirmed diagnoses, including 42 with HB and 28 with FNH, and 51 were diagnosed according to the clinical-imaging reference standard, including 37 with HB and 14 with FNH.

In the reader study, 41 cases in the temporal test cohort were initially marked by the junior radiologist as atypical or diagnostically uncertain, representing 33.9% of the test cohort. Baseline patient characteristics are summarized in **Table 1**. Compared with the training cohort, the temporal test cohort contained a higher proportion of FNH, a higher pathological confirmation rate, and older patients. The temporal test cohort also included a higher proportion of examinations performed on the Revolution CT scanner than the training cohort (79.3% vs. 56.2%, *p* < 0.001).

**Table 1:** Patient characteristics of the training and temporal test cohorts.

Characteristic	Overall (n = 612)	Training cohort (n = 491)	Temporal test cohort (n = 121)	*p* value
Diagnosis				<0.001
HB	523 (85.5)	444 (90.4)	79 (65.3)	
FNH	89 (14.5)	47 (9.6)	42 (34.7)	
Age, months, median (IQR)	22.0 (11.3–44.2)	20.2 (10.9–36.6)	42.2 (17.5–98.4)	<0.001
Age, years, median (IQR)	1.8 (0.9–3.7)	1.7 (0.9–3.1)	3.5 (1.5–8.2)	<0.001
Age group				<0.001
<5 years	499 (81.5)	423 (86.2)	76 (62.8)	
≥5 years	113 (18.5)	68 (13.8)	45 (37.2)	
Sex				0.359
Male	333 (54.4)	272 (55.4)	61 (50.4)	
Female	279 (45.6)	219 (44.6)	60 (49.6)	
CT scanner				<0.001
Discovery CT750 HD	240 (39.2)	215 (43.8)	25 (20.7)	
Revolution CT	372 (60.8)	276 (56.2)	96 (79.3)	
Reference standard				<0.001
Pathology-confirmed	237 (38.7)	167 (34.0)	70 (57.9)	
Clinical-imaging reference standard	375 (61.3)	324 (66.0)	51 (42.1)	
Pathology-confirmed HB	186/523 (35.6)	144/444 (32.4)	42/79 (53.2)	
Pathology-confirmed FNH	51/89 (57.3)	23/47 (48.9)	28/42 (66.7)	
Atypical cases identified by junior radiologist	—	—	41 (33.9)	—

The *p* values compare the training and temporal test cohorts. Continuous variables were compared using the Mann-Whitney U test; categorical variables were compared using the chi-square test or Fisher’s exact test, as appropriate. Atypical cases were defined only for the reader study in the temporal test cohort and indicate lesions considered atypical or diagnostically uncertain by the junior radiologist. HB, hepatoblastoma; FNH, focal nodular hyperplasia; IQR, interquartile range.

### 3.2 Diagnostic performance of DL, QEF, and DL+QEF models

In the temporally independent test cohort, DL-PVP showed the highest discrimination among the single-phase DL models, with an AUC of 0.932, exceeding those of DL-PCP and DL-AP (**Table 2**). The multiphasic DL ensemble, DL-3Phase, had an AUC of 0.936, an accuracy of 87.6%, a sensitivity of 94.9%, and an FNH specificity of 73.8%. The QEF model had an AUC of 0.955, an accuracy of 86.8%, an HB sensitivity of 82.3%, and an FNH specificity of 95.2%.

**Table 2:** Diagnostic performance of the models in the temporal test cohort.

Model	AUC (95% CI)	Accuracy, % (95% CI)	HB sensitivity, % (95% CI)	FNH specificity, % (95% CI)	Balanced accuracy, % (95% CI)	PPV, % (95% CI)	NPV, % (95% CI)
DL-PCP	0.867 (0.803–0.925)	80.2 (72.2–86.3)	89.9 (81.3–94.8)	61.9 (46.8–75.0)	75.9 (67.6–83.7)	81.6 (72.2–88.4)	76.5 (60.0–87.6)
DL-AP	0.900 (0.831–0.961)	86.8 (79.6–91.7)	93.7 (86.0–97.3)	73.8 (58.9–84.7)	83.7 (76.4–90.8)	87.1 (78.3–92.6)	86.1 (71.3–93.9)
DL-PVP	0.932 (0.883–0.971)	85.1 (77.7–90.4)	96.2 (89.4–98.7)	64.3 (49.2–77.0)	80.2 (72.5–87.9)	83.5 (74.6–89.7)	90.0 (74.4–96.5)
DL-3Phase	0.936 (0.891–0.973)	87.6 (80.6–92.3)	94.9 (87.7–98.0)	73.8 (58.9–84.7)	84.4 (76.7–91.0)	87.2 (78.5–92.7)	88.6 (74.0–95.5)
QEF	0.955 (0.914–0.988)	86.8 (79.6–91.7)	82.3 (72.4–89.1)	95.2 (84.2–98.7)	88.8 (83.1–93.7)	97.0 (89.8–99.2)	74.1 (61.1–83.9)
DL+QEF	0.980 (0.956–0.996)	94.2 (88.5–97.2)	94.9 (87.7–98.0)	92.9 (81.0–97.5)	93.9 (89.0–98.1)	96.2 (89.3–98.7)	90.7 (78.4–96.3)

HB was defined as the positive class and FNH as the negative class. Sensitivity refers to HB sensitivity, and specificity refers to FNH specificity. DL, deep learning model; QEF, quantitative enhancement feature model; AUC, area under the receiver operating characteristic curve; PPV, positive predictive value; NPV, negative predictive value.

**Figure 3:**
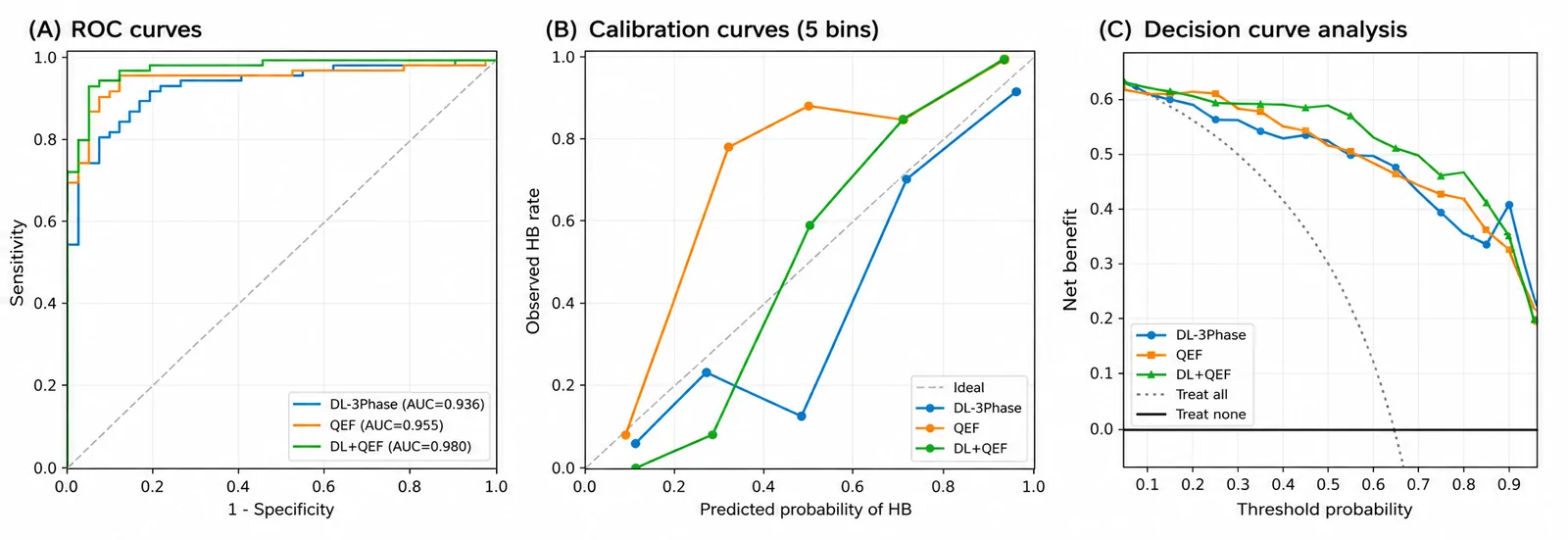
Diagnostic performance of DL-3Phase, QEF, and DL+QEF models in the temporal test cohort. **(A)** Receiver operating characteristic curves for DL-3Phase, QEF, and DL+QEF. **(B)** Calibration curves using five probability bins. The dashed diagonal line indicates perfect calibration. **(C)** Decision curve analysis across threshold probabilities. The dotted gray curve indicates the treat-all strategy, and the solid black line indicates the treat-none strategy. HB was considered the positive class. DL-3Phase denotes unweighted probability averaging of the precontrast, arterial, and portal venous phase DL models. QEF, quantitative enhancement feature model based on manual small-ROI attenuation measurements. DL+QEF denotes unweighted probability averaging of DL-3Phase and QEF. All analyses were performed in the locked temporal test cohort of 121 patients, including 79 with HB and 42 with FNH.

The DL+QEF fusion model had an AUC of 0.980 (bootstrap 95% CI, 0.956–0.996), with an accuracy of 94.2%, an HB sensitivity of 94.9%, an FNH specificity of 92.9%, a positive predictive value of 96.2%, and a negative predictive value of 90.7% (**Table 2**; **Figure 3A**).

The standardized coefficients of the QEF model showed that the features with larger absolute standardized coefficients included the wash-in ratio, precontrast tumor attenuation, arterial- and portal venous-phase vessel-normalized enhancement indices, the portal venous-phase tumor-to-liver attenuation difference, and washout-related indices (**Supplementary Figure 1**). In the temporally independent test cohort, FNH had higher precontrast, arterial-phase, and portal venous-phase tumor attenuation than HB, as well as higher arterial- and portal venous-phase tumor-to-liver ratios. The wash-in ratio also tended to be higher in FNH, although this difference was not statistically significant (**Supplementary Figure 2**). Multiphasic CT attenuation profiles and scatter plots demonstrated distinct overall enhancement patterns between the two groups, with partial overlap at the individual-case level (**Supplementary Figures 3**, **4**).

### 3.3 Comparative performance, calibration, and decision curve analysis

For the prespecified primary comparison, DL+QEF had a higher AUC than DL-3Phase (paired AUC difference, 0.043; 95% CI, 0.015–0.077; DeLong p = 0.005). In secondary comparisons, DL+QEF also had a higher AUC than DL-PVP (paired AUC difference, 0.047; 95% CI, 0.016–0.086; nominal DeLong p = 0.007), whereas the AUC difference between DL+QEF and QEF was smaller and uncertain (0.024; 95% CI, −0.007 to 0.062; nominal DeLong p = 0.163). The corresponding paired AUC differences were 0.043 (95% CI, 0.015–0.077) versus DL-3Phase, 0.047 (95% CI, 0.016–0.086) versus DL-PVP, and 0.024 (95% CI, −0.007 to 0.062) versus QEF.

At the prespecified classification threshold of 0.50, DL+QEF improved FNH specificity relative to DL-3Phase while preserving HB sensitivity. HB sensitivity was 94.9% for both models, whereas FNH specificity was higher for DL+QEF than for DL-3Phase (92.9% vs. 73.8%; exact McNemar *p* = 0.008). Compared with QEF, DL+QEF increased accuracy from 86.8% to 94.2% and HB sensitivity from 82.3% to 94.9%, with a slight reduction in FNH specificity from 95.2% to 92.9%; the paired classifications differed (exact McNemar *p* = 0.022).

In the calibration analysis, DL+QEF had a Brier score of 0.069, lower than those of DL-3Phase (0.101) and QEF (0.090). The calibration intercept, calibration slope, and expected calibration error of DL+QEF were 0.121, 2.513, and 0.121, respectively, indicating that the predicted probabilities were not fully calibrated despite the comparatively low Brier score. The calibration curve showed an overall correspondence between predicted probabilities and observed HB proportions, although deviations were present across probability intervals (**Figure 3B**). Decision curve analysis showed that DL+QEF provided a positive net benefit over both the treat-all and treat-none strategies across the main range of threshold probabilities (**Figure 3C**).

### 3.4 Reference-standard sensitivity analysis

To assess the influence of reference standard heterogeneity on model performance, sensitivity analyses were performed in subsets defined by the reference standard. In the pathology-confirmed test subset, which included 70 patients with 42 HB and 28 FNH, DL+QEF had an AUC of 0.972, an accuracy of 92.9%, an HB sensitivity of 95.2%, and an FNH specificity of 89.3%. In the clinical-imaging reference standard subset, DL+QEF had an AUC of 0.992, an accuracy of 96.1%, an HB sensitivity of 94.6%, and an FNH specificity of 100.0% (**Table 3**). Within the pathology-confirmed subset, the AUC of DL+QEF remained higher than that of DL-3Phase (*p* = 0.031). Overall, DL+QEF showed broadly consistent performance across subsets defined by different reference standards. In the clinical-imaging reference standard subset, the 100.0% FNH specificity was based on 14 FNH cases.

**Table 3:** Sensitivity analysis according to the reference standard.

Subset	n	HB/FNH	AUC (95% CI)	Accuracy, % (95% CI)	HB sensitivity, % (95% CI)	FNH specificity, % (95% CI)	Balanced accuracy, % (95% CI)	PPV, % (95% CI)	NPV, % (95% CI)
Overall temporal test cohort	121	79/42	0.980 (0.956–0.996)	94.2 (88.5–97.2)	94.9 (87.7–98.0)	92.9 (81.0–97.5)	93.9 (89.0–98.1)	96.2 (89.3–98.7)	90.7 (78.4–96.3)
Pathology-confirmed subset	70	42/28	0.972 (0.934–0.998)	92.9 (84.3–96.9)	95.2 (84.2–98.7)	89.3 (72.8–96.3)	92.3 (85.1–98.2)	93.0 (81.4–97.6)	92.6 (76.6–97.9)
Clinical-imaging reference subset	51	37/14	0.992 (0.971–1.000)	96.1 (86.8–98.9)	94.6 (82.3–98.5)	100.0 (78.5–100.0)	97.3 (93.2–100.0)	100.0 (90.1–100.0)	87.5 (64.0–96.5)

Pathology-confirmed cases were confirmed by surgery or biopsy. Clinical-imaging reference cases were diagnosed on the basis of clinical records, imaging findings, available laboratory information, documented multidisciplinary assessment, and/or longitudinal follow-up. DL, deep learning model; QEF, quantitative enhancement feature model; AUC, area under the receiver operating characteristic curve; PPV, positive predictive value; NPV, negative predictive value.

### 3.5 Reader study in the full temporal test cohort

**Figure 4:**
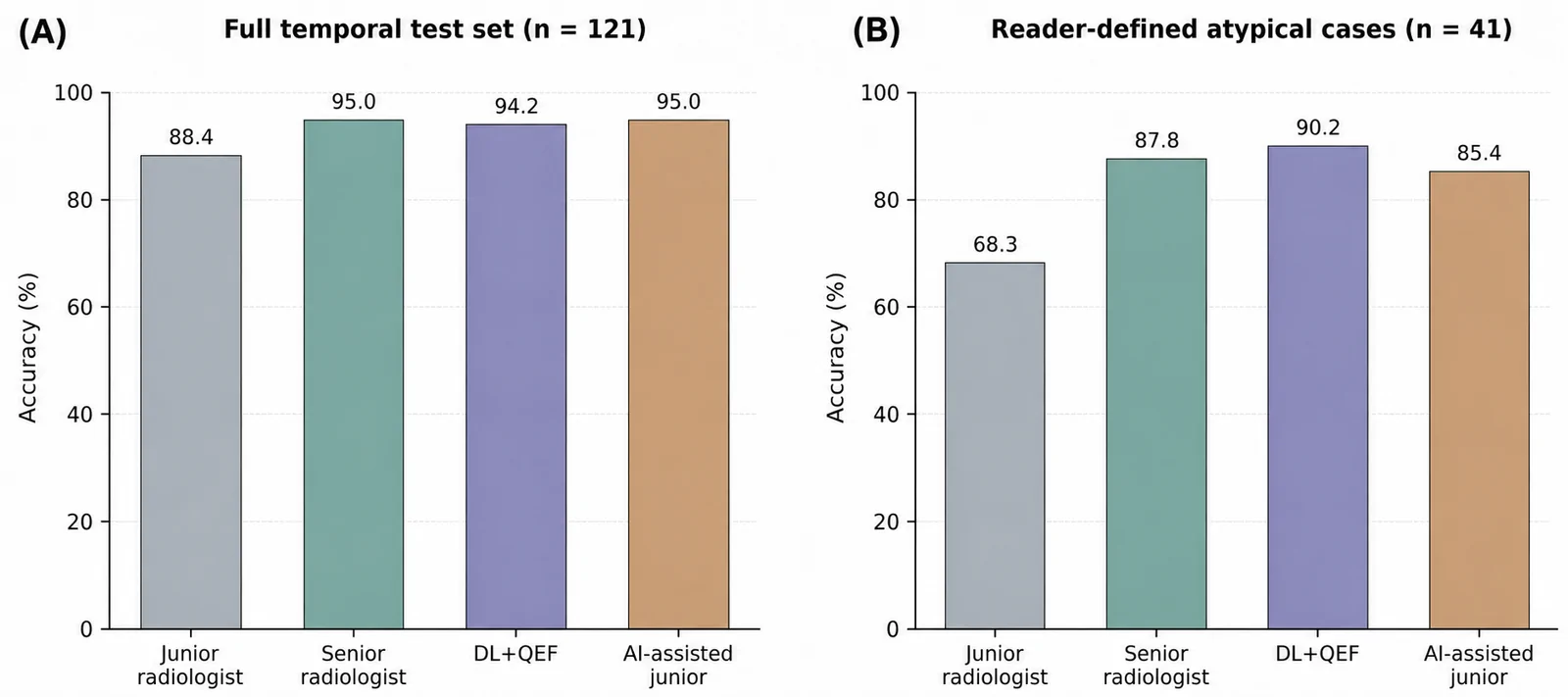
Reader study performance in the temporal test cohort. Bar charts show diagnostic accuracy of the junior radiologist, senior radiologist, DL+QEF model, and AI-assisted junior radiologist in the full temporal test cohort **(A)** and reader-defined atypical cases **(B)**. DL+QEF denotes the fusion model combining DL-3Phase and QEF probabilities.

In the full temporal test cohort, the junior radiologist had lower accuracy than the senior radiologist (88.4% vs. 95.0%; McNemar *p* = 0.0215), with an HB sensitivity of 89.9% and an FNH specificity of 85.7%, compared with 96.2% and 92.9%, respectively, for the senior radiologist (**Table 4**; **Figure 4A**). At the case level, DL+QEF and the unaided junior radiologist disagreed in 15 cases; DL+QEF was correct in 11, whereas the junior radiologist was correct in 4 (**Supplementary Table S1**). During the model-assisted session, the junior radiologist achieved an accuracy of 95.0%, with an HB sensitivity of 94.9% and an FNH specificity of 95.2%. Among the 14 cases misclassified during the initial unaided session, 8 were correctly classified during the assisted session, whereas none of the 107 cases correctly classified during the initial session were misclassified during the assisted session (McNemar *p* = 0.0078). Pairwise comparisons showed no significant difference between DL+QEF and either the senior radiologist or the AI-assisted junior radiologist in the full temporal test cohort.

**Table 4:** Reader and model performance in the full temporal test cohort.

Method	AUC (95% CI)	Accuracy, % (95% CI)	HB sensitivity, % (95% CI)	FNH specificity, % (95% CI)	Balanced accuracy, % (95% CI)	PPV, % (95% CI)	NPV, % (95% CI)	Accuracy difference vs. DL+QEF, percentage points (95% CI)	*p* value vs. DL+QEF
Junior radiologist	NA	88.4 (81.5–93.0)	89.9 (81.3–94.8)	85.7 (72.2–93.3)	87.8 (81.2–93.7)	92.2 (84.0–96.4)	81.8 (68.0–90.5)	−5.8 (−11.6 to 0.8)	0.118
Senior radiologist	NA	95.0 (89.6–97.7)	96.2 (89.4–98.7)	92.9 (81.0–97.5)	94.5 (89.6–98.7)	96.2 (89.4–98.7)	92.9 (81.0–97.5)	0.8 (−3.3 to 5.0)	1.000
DL+QEF	0.980 (0.956–0.996)	94.2 (88.5–97.2)	94.9 (87.7–98.0)	92.9 (81.0–97.5)	93.9 (89.0–98.1)	96.2 (89.3–98.7)	90.7 (78.4–96.3)	Reference	Reference
AI-assisted junior radiologist	NA	95.0 (89.6–97.7)	94.9 (87.7–98.0)	95.2 (84.2–98.7)	95.1 (90.3–98.7)	97.4 (91.0–99.3)	90.9 (78.8–96.4)	0.8 (−3.3 to 5.0)	1.000

The *p* values were calculated using exact McNemar tests comparing each method with DL+QEF. DL, deep learning model; QEF, quantitative enhancement feature model; AUC, area under the receiver operating characteristic curve; PPV, positive predictive value; NPV, negative predictive value.

### 3.6 Performance in reader-defined atypical cases

As an exploratory analysis, diagnostic performance was evaluated in the reader-defined atypical subset, which included 41 patients (30 with HB and 11 with FNH). At the fixed probability threshold of 0.50, QEF achieved an AUC of 0.915, an accuracy of 75.6%, an HB sensitivity of 70.0%, and an FNH specificity of 90.9%. The corresponding values for DL+QEF were 0.939, 90.2%, 90.0%, and 90.9%, respectively. Although the AUC of DL+QEF was numerically higher than that of QEF, the difference was not statistically significant (DeLong *p* = 0.416; paired AUC difference, 0.024 [95% CI, −0.030 to 0.094]). At the fixed threshold of 0.50, the paired classifications differed (exact McNemar *p* = 0.031); DL+QEF correctly classified six cases misclassified by QEF, whereas no case showed the opposite pattern.

The junior radiologist achieved an accuracy of 68.3%, an HB sensitivity of 73.3%, and an FNH specificity of 54.5%. DL+QEF achieved higher accuracy than the junior radiologist in this subset (90.2% vs. 68.3%; exact McNemar *p* = 0.012). In this subset, DL+QEF and the unaided junior radiologist disagreed in 11 cases; DL+QEF was correct in 10, whereas the junior radiologist was correct in 1 (**Supplementary Table S1**). The senior radiologist and the AI-assisted junior radiologist achieved accuracies of 87.8% and 85.4%, respectively (**Table 5**; **Figure 4B**). During the assisted-reading session, 7 of the 13 cases misclassified during the initial unaided session were correctly classified, whereas none of the 28 cases correctly classified during the initial session was misclassified during the assisted session. Accordingly, junior-radiologist accuracy was 68.3% during unaided reading and 85.4% during assisted reading (exact McNemar *p* = 0.0156). The senior radiologist also achieved higher accuracy than the junior radiologist (exact McNemar *p* = 0.0078). No significant difference was observed between DL+QEF and either the senior radiologist or the AI-assisted junior radiologist.

**Table 5:** Reader and model performance in reader-defined atypical cases.

Method	AUC (95% CI)	Accuracy, % (95% CI)	HB sensitivity, % (95% CI)	FNH specificity, % (95% CI)	Balanced accuracy, % (95% CI)	PPV, % (95% CI)	NPV, % (95% CI)	Accuracy difference vs. DL+QEF, percentage points (95% CI)	*p* value vs. DL+QEF
Junior radiologist	NA	68.3 (53.0–80.4)	73.3 (55.6–85.8)	54.5 (28.0–78.7)	63.9 (47.0–80.9)	81.5 (63.3–91.8)	42.9 (21.4–67.4)	−22.0 (−36.6 to −7.3)	0.012
Senior radiologist	NA	87.8 (74.5–94.7)	90.0 (74.4–96.5)	81.8 (52.3–94.9)	85.9 (71.8–96.7)	93.1 (78.0–98.1)	75.0 (46.8–91.1)	−2.4 (−12.2 to 7.3)	1.000
QEF	0.915 (0.803–0.994)	75.6 (60.7–86.2)	70.0 (52.1–83.3)	90.9 (62.3–98.4)	80.5 (68.0–91.7)	95.5 (78.2–99.2)	52.6 (31.7–72.7)	−14.6 (−26.8 to −4.9)	0.031
DL+QEF	0.939 (0.833–1.000)	90.2 (77.5–96.1)	90.0 (74.4–96.5)	90.9 (62.3–98.4)	90.5 (78.5–98.3)	96.4 (82.3–99.4)	76.9 (49.7–91.8)	Reference	Reference
AI-assisted junior radiologist	NA	85.4 (71.6–93.1)	86.7 (70.3–94.7)	81.8 (52.3–94.9)	84.2 (70.2–95.5)	92.9 (77.4–98.0)	69.2 (42.4–87.3)	−4.9 (−14.6 to 4.9)	0.625

Atypical cases were defined by the junior radiologist during independent unaided reading as lesions with atypical imaging appearance or diagnostic uncertainty. The *p* values were calculated using exact McNemar tests comparing each method with DL+QEF. DL, deep learning model; QEF, quantitative enhancement feature model; AUC, area under the receiver operating characteristic curve; PPV, positive predictive value; NPV, negative predictive value.

### 3.7 Subgroup, sensitivity, and exploratory analyses

Manual tumor attenuation measurements showed good-to-excellent interobserver agreement, with ICCs of 0.805 (95% CI, 0.775–0.831), 0.900 (95% CI, 0.884–0.914), and 0.929 (95% CI, 0.918–0.939) for the precontrast, arterial, and portal venous phases, respectively. In the temporally independent test cohort, the WT-QEF model constructed from whole-tumor mean attenuation had an AUC of 0.965, an accuracy of 93.4%, an HB sensitivity of 92.4%, and an FNH specificity of 95.2%. The predicted probabilities of QEF and WT-QEF were strongly correlated, with a Pearson r of 0.846 and a Spearman ρ of 0.833, and the classification agreement at the fixed threshold of 0.50 was 90.9%. The DL+WT-QEF model, which fused DL-3Phase with WT-QEF, had an AUC of 0.975, an accuracy of 94.2%, an HB sensitivity of 94.9%, and an FNH specificity of 92.9%. Its AUC did not differ significantly from that of DL+QEF (DeLong *p* = 0.888). Using a probability threshold of 0.50, the logistic regression-based late-fusion model fitted to the training-cohort out-of-fold probabilities achieved an AUC of 0.964, an accuracy of 90.9%, an HB sensitivity of 96.2%, and an FNH specificity of 81.0%, and therefore did not improve on the prespecified probability-averaging strategy.

Using the composite quantitative hyperenhancement definition, 72 of 121 cases (59.5%) were classified as hyperenhancing. Hyperenhancement showed only partial overlap with reader-defined atypicality: 25 of the 72 hyperenhancing cases (34.7%) were categorized as atypical or diagnostically uncertain, accounting for 25 of the 41 reader-defined atypical cases (61.0%). Junior-reader accuracy was similar between hyperenhancing and non-hyperenhancing cases (87.5% [63/72] vs. 89.8% [44/49]; Fisher’s exact test, *p* = 0.779). Under the stricter 90th-percentile-based definition of marked hyperenhancement, 29 cases (24.0%) were identified. Junior-reader accuracy was lower in this subgroup than in the remaining cases, although the difference did not reach statistical significance (79.3% [23/29] vs. 91.3% [84/92]; Fisher’s exact test, *p* = 0.098).

In age-stratified analyses, DL+QEF achieved an AUC of 0.992, an accuracy of 96.1%, an HB sensitivity of 95.5%, and an FNH specificity of 100.0% among patients younger than 5 years (n = 76; 66 HB and 10 FNH). Among patients aged 5 years or older (n = 45; 13 HB and 32 FNH), the corresponding values were 0.971, 91.1%, 92.3%, and 90.6%, respectively. In sex-stratified analyses, the AUC, accuracy, HB sensitivity, and FNH specificity were 0.999, 98.4%, 97.8%, and 100.0%, respectively, in male patients (n = 61; 46 HB and 15 FNH), and 0.965, 90.0%, 90.9%, and 88.9%, respectively, in female patients (n = 60; 33 HB and 27 FNH) (**Supplementary Table S2**). Scanner type was not significantly associated with diagnosis within either the training or temporal test cohort. In the temporal test cohort, DL+QEF achieved an AUC of 0.981, an accuracy of 92.0%, an HB sensitivity of 92.9%, and an FNH specificity of 90.9% on Discovery CT750 HD examinations (n = 25; 14 HB and 11 FNH). The corresponding values on Revolution examinations were 0.980, 94.8%, 95.4%, and 93.5%, respectively (n = 96; 65 HB and 31 FNH) (**Supplementary Table S3**).

In the threshold sensitivity analysis, the model-specific training-cohort out-of-fold Youden thresholds were 0.168, 0.288, 0.797, 0.450, 0.397, and 0.455 for DL-PCP, DL-AP, DL-PVP, DL-3Phase, QEF, and DL+QEF, respectively. Using its training-derived cutoff, DL+QEF achieved an accuracy of 92.6%, an HB sensitivity of 97.5%, and an FNH specificity of 83.3% in the temporal test cohort (**Supplementary Table S4**).

## 4 Discussion

### 4.1 Key findings

In this study, we developed and validated an artificial intelligence model based on pediatric multiphasic contrast-enhanced CT for differentiating hepatoblastoma from focal nodular hyperplasia. In the temporally independent test cohort, the DL+QEF model showed favorable diagnostic performance by integrating deep learning-derived image features with quantitative features reflecting multiphasic CT enhancement patterns. In the reader study, the junior radiologist achieved higher accuracy during the model-assisted session than during the initial unaided session, suggesting that model-derived probability estimates may provide useful decision support for less experienced readers during CT interpretation.

### 4.2 Clinical context and implications

Given its reliance on manual tumor segmentation and ROI measurements, the current workflow should be regarded as an expert-assisted proof-of-concept decision-support tool for cases in which HB and FNH constitute the principal differential diagnostic considerations. Its intended role is to inform subsequent laboratory evaluation, MRI assessment, tissue sampling, or multidisciplinary discussion rather than to serve as a stand-alone basis for final diagnosis. Because multiphasic CT involves ionizing radiation, the model should be applied only to clinically indicated pretreatment CT examinations and should not prompt additional phases or repeat examinations. MRI remains an important complementary modality, particularly when FNH is suspected.

### 4.3 Comparison with similar research

Previous liver AI studies have shown that deep learning and radiomics can support focal liver lesion detection, benign-malignant classification, and subtype differentiation (29, 30). However, most existing liver AI models have focused on adult liver lesions (30, 31) and cannot be directly extrapolated to pediatric HB–FNH differentiation because of differences in disease spectrum, background liver status, enhancement patterns, and clinical pathways (21, 22). Existing pediatric liver AI studies have mainly addressed HB risk stratification or differentiation between HB and hepatic hemangioma in early infancy (32, 33). In contrast, CT-based differentiation of pediatric HB from FNH remains insufficiently studied.

### 4.4 Explanations of findings

DL-3Phase and QEF showed distinct performance profiles in the test cohort. DL-3Phase maintained high sensitivity for HB but had relatively limited specificity for FNH, whereas QEF showed high FNH specificity but lower HB sensitivity than the fusion model. DL+QEF provided a more balanced sensitivity-specificity profile, supporting the complementary value of multiphasic CT-based DL predictions and enhancement-related quantitative information. Deep learning may capture lesion morphology, internal heterogeneity, and multiphasic enhancement patterns, whereas QEF provides an interpretable quantification of phase-specific attenuation changes corresponding to the routine radiologic assessment of lesion vascularity and enhancement kinetics. This complementary information may provide a relatively interpretable basis for the fusion model.

Because enhancement features were derived from manual ROI measurements, ROI placement may be influenced by observer experience, lesion heterogeneity, and slice selection (34, 35). The two radiologists showed good-to-excellent agreement for CT attenuation measurements, supporting the reliability of the manual ROI-based measurements. In addition, both WT-QEF and DL+WT-QEF, which were constructed using whole-tumor mean attenuation, maintained favorable diagnostic performance, and the predicted probabilities of QEF and WT-QEF were strongly correlated. These findings suggest that the diagnostic value of enhancement features was not entirely dependent on local ROI placement. Nevertheless, whole-tumor mean attenuation should not be regarded as a direct substitute for standardized small-ROI measurements, because these two strategies reflect related but non-identical imaging information. Whole-tumor measurements summarize average enhancement across the entire lesion, whereas small-ROI measurements target representative solid enhancing areas.

From an implementation perspective, QEF may be easier to translate into routine practice because it can be derived from standardized small-ROI attenuation measurements without whole-tumor segmentation or a dedicated DL pipeline. In the reader-defined atypical subset, DL+QEF showed a numerically higher AUC than QEF, although the difference in AUC was not statistically significant. At the fixed threshold of 0.50, however, DL+QEF achieved higher accuracy and HB sensitivity while maintaining the same FNH specificity, and the paired classifications differed (exact McNemar *p* = 0.031). These findings suggest that the DL component may provide complementary diagnostic information in challenging cases. However, this potential benefit should be weighed against the additional requirements for whole-tumor segmentation and model processing, as well as the simpler implementation of the QEF approach.

Exploratory analyses further suggested that HB–FNH differentiation cannot be adequately characterized by threshold-based enhancement phenotypes alone. The composite quantitative hyperenhancement phenotype showed only partial overlap with reader-defined atypicality and was not associated with significantly lower junior-reader accuracy. Although HB and FNH showed different group-level enhancement patterns (**Supplementary Figure 3**), their arterial- and portal venous-phase attenuation values partially overlapped at the individual-case level (**Supplementary Figure 4**), indicating that group-level differences do not necessarily provide reliable separation in individual patients. Therefore, the value of the model lies not in deriving a single attenuation threshold, but in integrating multiple partially overlapping imaging features across CT phases to provide adjunctive support when imaging findings are not fully concordant in individual cases.

### 4.5 Reader study and clinical use

The junior radiologist achieved relatively high accuracy in the full temporal test cohort but substantially lower performance in the reader-defined atypical subset, indicating that these cases were particularly challenging for that reader. During the model-assisted session, junior-radiologist accuracy was higher in both the full cohort and the atypical subset and approached that of the senior radiologist, suggesting potential decision-support value when imaging appearances overlap or reader confidence is limited (31, 36–38). However, because atypicality was defined subjectively by the same junior radiologist and only one junior radiologist participated in the sequential assisted-reading experiment, these findings should be considered exploratory. Larger multireader, multicase studies using objective or consensus-based definitions of difficult cases are needed.

### 4.6 Strengths and limitations

A methodological strength of this study was the use of a temporally independent test cohort rather than a random split, providing a more clinically relevant assessment of temporal generalizability (39).

This study has several limitations. First, this was a single-center retrospective study. Although the cohort was relatively large for this uncommon pediatric differential diagnosis, the number of FNH cases remained limited, particularly in the training cohort and several subgroup analyses, reducing the precision of FNH-specific estimates. The proportion of FNH and scanner distribution also differed between the training and temporal test cohorts, potentially reflecting temporal changes in referral patterns and diagnostic recognition. Scanner type showed only weak associations with diagnosis, and scanner-stratified analyses demonstrated comparable DL+QEF performance; nevertheless, residual scanner-related effects cannot be excluded given the limited number of Discovery CT750 HD examinations in the temporal test cohort. Multicenter external validation across institutions, vendors, and imaging protocols is warranted (40). Second, the reference standard was heterogeneous. In routine pediatric liver tumor practice, pathological confirmation is not always available for every lesion, and some cases with typical clinical, laboratory, and imaging findings may be diagnosed through multidisciplinary consensus (5, 25). Although this design reflects real-world diagnostic pathways and DL+QEF maintained favorable performance in the pathology-confirmed subset, pathological confirmation was not randomly available across the study population, and incorporation and differential-verification bias could not be fully excluded (41, 42). The 100.0% FNH specificity observed in the clinical-imaging reference standard subset should be interpreted cautiously because it was based on only 14 FNH cases. Third, AFP and other clinical variables were not included in the model. This design was intended to evaluate the diagnostic contribution of CT imaging features and to reduce potential incorporation bias arising from the use of clinical variables in establishing the reference diagnosis for some non-pathologically confirmed cases. Although DL+QEF showed favorable performance across age and sex subgroups, these analyses were exploratory and were not designed for formal between-subgroup comparisons. The precision of some subgroup estimates was also limited by the small number of FNH cases, including only 10 among patients younger than 5 years and 15 among male patients. Future studies should therefore compare CT-only AI models with models that integrate CT imaging features, laboratory findings, and other clinical information.

Fourth, this study relied on manual tumor segmentation and manual ROI measurements for QEF, which may limit workflow efficiency and reproducibility. Annotation, ROI-measurement, and model-processing times were not prospectively recorded and therefore could not be reliably quantified. Independent duplicate segmentation was not performed, precluding formal assessment of interobserver segmentation agreement, although all VOIs underwent senior radiologist review. Future studies should explore automatic or semi-automatic segmentation and standardized strategies for automated ROI placement to improve clinical usability. Fifth, the reader study included only two radiologists, and only one junior radiologist participated in the sequential assisted-reading session, limiting causal inference and the generalizability of the findings. Recall, learning, or increased familiarity with the cases could not be completely excluded despite the 4-week washout period and rerandomization. Further multireader, multicase studies with counterbalanced reading orders should include readers from different institutions with varying levels of experience and expertise in pediatric abdominal imaging.

## 5 Conclusion

In conclusion, the DL+QEF model showed favorable performance for differentiating pediatric HB from FNH in a temporally independent test cohort. DL-derived multiphasic imaging features and QEF-based quantitative enhancement information provided complementary value, and the model showed potential to support junior-radiologist interpretation. This model may serve as an adjunctive decision-support tool during initial CT assessment when HB and FNH constitute the principal differential diagnostic considerations, particularly in cases with overlapping enhancement patterns or limited diagnostic confidence. Multicenter external validation and prospective studies are needed to confirm its clinical value.

## Data Availability

The data supporting the findings of this study are not publicly available due to patient privacy and institutional data protection requirements. De-identified data may be made available from the corresponding author upon reasonable request and subject to approval by the relevant institutional authorities.
