# Supplementary material for: A pretreatment multiphasic CT-based decision-support model for differentiating pediatric hepatoblastoma from focal nodular hyperplasia
Source: Front Oncol. 2026 Aug 24;16:1923907. doi: 10.3389/fonc.2026.1923907 (PMC13547000; doi:10.3389/fonc.2026.1923907)
Supplement: Supplementary file 1 [file SupplementaryFile1.docx]

Supplementary Figure 1. Standardized coefficients of the QEF model. Horizontal bar plot showing the standardized coefficients from the logistic regression-based QEF. HB was defined as the positive class. Positive coefficients indicate that higher feature values increased the predicted probability of HB, whereas negative coefficients indicate that higher feature values favored FNH. Features were ranked according to the absolute magnitude of their standardized coefficients. Because quantitative enhancement features may be correlated, the coefficients should be interpreted as model-level associations rather than independent causal effects. QEF = quantitative enhancement feature model; HB = hepatoblastoma; FNH = focal nodular hyperplasia; PCP = precontrast phase; AP = arterial phase; PVP = portal venous phase.

Supplementary Figure 2. Distribution of clinically interpretable quantitative enhancement features in the temporal test cohort. Violin plots with overlaid boxplots and individual data points show the distributions of six clinically interpretable quantitative enhancement features in HB and FNH: tumor attenuation in the PCP, AP and PVP; tumor-to-liver attenuation ratios in AP and PVP; and the wash-in ratio. Boxplots show medians and interquartile ranges, and each point represents an individual case. The p values were calculated using the two-sided Mann-Whitney U test. HB = hepatoblastoma; FNH = focal nodular hyperplasia; PCP = precontrast phase; AP = arterial phase; PVP = portal venous phase.

Supplementary Figure 3. Multiphasic enhancement patterns of HB and FNH in the temporal test cohort. (A) Absolute tumor attenuation curves. Median tumor attenuation values across the PCP, AP, and PVP are shown for HB and FNH, with shaded areas indicating interquartile ranges. This panel reflects both baseline precontrast attenuation and contrast-enhanced attenuation across phases. (B) Tumor-to-liver ratio curves. Median tumor-to-liver attenuation ratios across the PCP, AP, and PVP are shown for HB and FNH, with shaded areas indicating interquartile ranges. Ratios were calculated as tumor attenuation divided by liver attenuation in the same phase. This panel shows lesion enhancement relative to the liver background and compares multiphasic enhancement patterns between HB and FNH. HB, hepatoblastoma; FNH, focal nodular hyperplasia; PCP, precontrast phase; AP, arterial phase; PVP, portal venous phase.

Supplementary Figure 4. Scatter plot of arterial- and portal venous-phase tumor attenuation. Scatter plot showing arterial- and portal venous-phase tumor attenuation values in the temporal test cohort. FNH showed generally higher arterial- and portal venous-phase tumor attenuation than HB, although the distributions partially overlapped at the individual-case level. The shaded region indicates the two-dimensional overlap zone. This figure was used only for exploratory interpretation and was not used for model training, feature selection, or threshold determination. HB, hepatoblastoma; FNH, focal nodular hyperplasia; AP, arterial phase; PVP, portal venous phase.
