# Supplementary material for: A pretreatment multiphasic CT-based decision-support model for differentiating pediatric hepatoblastoma from focal nodular hyperplasia
Source: Front Oncol. 2026 Aug 24;16:1923907. doi: 10.3389/fonc.2026.1923907 (PMC13547000; doi:10.3389/fonc.2026.1923907)
Supplement: Supplementary file 2 [file SupplementaryFile2.docx]

**Supplementary Methods S1. Quantitative Enhancement Feature Definitions, Preprocessing, and Final Model Specification**

**Attenuation measurements and averaging.** Two radiologists independently performed phase-specific CT attenuation measurements using the same predefined small-ROI placement rules. For each phase, each radiologist placed two ROIs in representative solid tumor regions, and the two measurements were averaged to obtain a reader-specific mean attenuation value. For QEF calculation, the reader-specific mean values from the two radiologists were subsequently averaged to obtain the final phase-specific tumor attenuation value. Interobserver agreement was assessed using the reader-specific mean attenuation values obtained by each radiologist:

$$\overline{T}_{r,p} = \frac{{ROI}_{r,p,1} + {ROI}_{r,p,2}}{2}$$

$$T_{p} = \frac{\overline{T}_{1,p} + \overline{T}_{2,p}}{2}$$

**Notation.** r denotes the radiologist and p denotes the CT phase. The reader-specific mean attenuation value is denoted by T̄r,p, and Tp denotes the final phase-specific tumor attenuation value averaged across the two radiologists. T_PCP, T_AP, and T_PVP denote the final tumor attenuation values on precontrast, arterial-phase, and portal venous-phase CT, respectively. L_PCP, L_AP, and L_PVP denote liver parenchymal attenuation in the corresponding phases. A_AP denotes abdominal aortic attenuation in the arterial phase, and PV_PVP denotes portal venous attenuation in the portal venous phase. Attenuation values and attenuation differences are expressed in Hounsfield units (HU); ratios and indices are dimensionless.

| **Feature** | **Code variable** | **Formula** | **Unit** | **Interpretation** |
| --- | --- | --- | --- | --- |
| Precontrast tumor attenuation | T_PCP_mean | $T_{PCP}$ | HU | Mean tumor attenuation on precontrast CT. |
| Arterial-phase tumor attenuation | T_AP_mean | $T_{AP}$ | HU | Mean tumor attenuation on arterial-phase CT. |
| Portal venous-phase tumor attenuation | T_PVP_mean | $T_{PVP}$ | HU | Mean tumor attenuation on portal venous-phase CT. |
| Arterial enhancement increment | delta_AP_PCP | $T_{AP} - T_{PCP}$ | HU | Absolute increase in tumor attenuation from the precontrast to arterial phase. |
| Portal venous enhancement increment | delta_PVP_PCP | $T_{PVP} - T_{PCP}$ | HU | Absolute increase in tumor attenuation from the precontrast to portal venous phase. |
| Arterial-to-portal venous attenuation change | delta_PVP_AP | $T_{PVP} - T_{AP}$ | HU | Change in tumor attenuation from the arterial to portal venous phase; positive values indicate continued enhancement. |
| Precontrast tumor-to-liver ratio | T_to_L_PCP | $\frac{T_{PCP}}{L_{PCP}}$ | Dimensionless | Tumor attenuation normalized to liver attenuation on precontrast CT. |
| Arterial-phase tumor-to-liver ratio | T_to_L_AP | $\frac{T_{AP}}{L_{AP}}$ | Dimensionless | Tumor attenuation normalized to liver attenuation in the arterial phase. |
| Portal venous-phase tumor-to-liver ratio | T_to_L_PVP | $\frac{T_{PVP}}{L_{PVP}}$ | Dimensionless | Tumor attenuation normalized to liver attenuation in the portal venous phase. |
| Arterial-phase tumor-to-liver difference | T_minus_L_AP | $T_{AP} - L_{AP}$ | HU | Difference between tumor and liver attenuation in the arterial phase. |
| Portal venous-phase tumor-to-liver difference | T_minus_L_PVP | $T_{PVP} - L_{PVP}$ | HU | Difference between tumor and liver attenuation in the portal venous phase. |
| Arterial-phase tumor-to-aorta ratio | T_to_A_AP | $\frac{T_{AP}}{A_{AP}}$ | Dimensionless | Tumor attenuation normalized to abdominal aortic attenuation in the arterial phase. |
| Portal venous-phase tumor-to-portal vein ratio | T_to_PV_PVP | $\frac{T_{PVP}}{{PV}_{PVP}}$ | Dimensionless | Tumor attenuation normalized to portal venous attenuation in the portal venous phase. |
| Wash-in ratio | wash_in_ratio | $\frac{T_{AP} - T_{PCP}}{T_{PCP}}$ | Dimensionless | Arterial enhancement relative to baseline tumor attenuation. |
| Washout index | washout_index | $\frac{T_{AP} - T_{PVP}}{\left( T_{AP} - T_{PCP} \right)}$ | Dimensionless | Arterial-to-portal venous attenuation decrease relative to the arterial enhancement increment; negative values indicate continued enhancement. |
| Relative clearance | relative_clearance | $\frac{T_{AP} - T_{PVP}}{T_{AP}}$ | Dimensionless | Arterial-to-portal venous attenuation decrease relative to arterial-phase tumor attenuation; negative values indicate continued enhancement. |

**Feature preprocessing and model fitting.** Continuous QEF features were standardized using z-score transformation before logistic regression modeling:

$$z = \frac{x - \mu_{training}}{\sigma_{training}}$$

During five-fold cross-validation, the feature mean and standard deviation were estimated within each training fold and applied to the corresponding held-out fold. For final model fitting, these parameters were re-estimated from the full 491-patient training cohort and applied unchanged to the temporal test cohort.

**Cross-validation and final model specification.** Missing feature values were imputed using the median. During five-fold cross-validation, the imputation and standardization parameters were estimated within each training fold and applied to the corresponding held-out fold to generate out-of-fold probabilities. For generation of out-of-fold probabilities, the regularization parameter was selected separately within each outer training fold by inner cross-validation using only the corresponding training partition, without access to the held-out fold. For final temporal test evaluation, the regularization parameter was selected by cross-validation within the full 491-patient training cohort and fixed before application to the temporal test cohort. After all out-of-fold predictions had been generated, the complete QEF pipeline was refitted using the full 491-patient training cohort and applied unchanged to the temporal test cohort.

The final model used L2-regularized logistic regression with the liblinear solver and balanced class weighting, incorporating 16 predictors and a regularization parameter C of 0.166810. HB and FNH were coded as 1 and 0, respectively. Balanced class weights were calculated as shown below, where n denotes the total training sample size and n_c denotes the number of training observations in class c.

$$w_{c}=\frac{n}{2n_{c}}$$

The final intercept was 1.736517. A fixed probability threshold of 0.500 was used for the primary analysis, whereas a model-specific threshold of 0.397408 selected by maximizing the Youden index using training-cohort out-of-fold predictions was evaluated in the threshold sensitivity analysis.

**Final preprocessing parameters and regression coefficients.** The table below provides the final imputation values, standardization parameters, and standardized coefficients.

| **Code variable** | **Imputation median** | **Standardization mean** | **Standardization scale** | **Standardized coefficient** |
| --- | --- | --- | --- | --- |
| T_PCP_mean | 47.720000 | 47.572088 | 8.042971 | −0.831542 |
| T_AP_mean | 85.475000 | 91.246578 | 28.514282 | 0.053317 |
| T_PVP_mean | 98.130000 | 101.350896 | 25.730933 | −0.052574 |
| delta_AP_PCP | 39.025000 | 43.674491 | 26.727781 | 0.307110 |
| delta_PVP_PCP | 50.312500 | 53.764343 | 22.362539 | 0.238726 |
| delta_PVP_AP | 11.700000 | 10.039022 | 19.737414 | −0.144826 |
| T_to_L_PCP | 0.782186 | 0.786967 | 0.142687 | −0.012251 |
| T_to_L_AP | 1.185194 | 1.236001 | 0.400602 | −0.158677 |
| T_to_L_PVP | 0.882955 | 0.880002 | 0.197271 | −0.134902 |
| T_minus_L_AP | 13.440000 | 15.882933 | 30.138971 | −0.061627 |
| T_minus_L_PVP | −13.672500 | −14.998722 | 24.323556 | −0.496821 |
| T_to_A_AP | 0.222675 | 0.236345 | 0.082832 | −0.640503 |
| T_to_PV_PVP | 0.582231 | 0.586022 | 0.138268 | −0.586095 |
| wash_in_ratio | 0.831952 | 0.943209 | 0.626323 | −1.349406 |
| washout_index | −0.355251 | −0.550482 | 1.138276 | 0.189851 |
| relative_clearance | −0.149557 | −0.144991 | 0.219520 | 0.476675 |

Coefficients correspond to standardized feature values. For feature x_j, z_j = (x_j − μ_j) / s_j, where μ_j and s_j denote the final training-derived standardization mean and scale, respectively. The predicted probability of HB was calculated as follows:

$$P\left( \mathrm{HB} \right)=\frac{1}{1+exp\left[ -\left( 1.736517+\sum_{j=1}^{16} \beta_{j}z_{j} \right) \right]}$$

HB, hepatoblastoma; FNH, focal nodular hyperplasia; QEF, quantitative enhancement feature model.
