# Supplementary material for: A pretreatment multiphasic CT-based decision-support model for differentiating pediatric hepatoblastoma from focal nodular hyperplasia
Source: Front Oncol. 2026 Aug 24;16:1923907. doi: 10.3389/fonc.2026.1923907 (PMC13547000; doi:10.3389/fonc.2026.1923907)
Supplement: Supplementary file 3 [file Table1.docx]

**Supplementary Table S1. Case-level concordance, discordance, and diagnostic transitions in the reader study**

**Panel A. Unaided junior radiologist versus DL+QEF**

| **Subset** | **Both correct** | **DL+QEF correct/ junior incorrect** | **Junior correct/ DL+QEF incorrect** | **Both incorrect** | **Total discordant** | **Exact McNemar p** |
| --- | --- | --- | --- | --- | --- | --- |
| Overall temporal test cohort (n = 121) | 103 | 11 | 4 | 3 | 15 | 0.118 |
| Reader-defined atypical subset (n = 41) | 27 | 10 | 1 | 3 | 11 | 0.0117 |

**Panel B. Unaided versus model-assisted junior-radiologist interpretation**

| **Subset** | **Correct in both sessions** | **Incorrect unaided/ correct assisted** | **Correct unaided/ incorrect assisted** | **Incorrect in both sessions** | **Changed classifications** | **Exact McNemar p** |
| --- | --- | --- | --- | --- | --- | --- |
| Overall temporal test cohort (n = 121) | 107 | 8 | 0 | 6 | 8 | 0.0078 |
| Reader-defined atypical subset (n = 41) | 28 | 7 | 0 | 6 | 7 | 0.0156 |

Note. Values are numbers of cases. Exact McNemar p values were calculated from the two discordant cells in each comparison. Reader-defined atypical cases were identified by the junior radiologist during the initial unaided reading session. DL+QEF denotes unweighted probability averaging of DL-3Phase and QEF, using the prespecified probability threshold of 0.50.
