# Supplementary material for: A pretreatment multiphasic CT-based decision-support model for differentiating pediatric hepatoblastoma from focal nodular hyperplasia
Source: Front Oncol. 2026 Aug 24;16:1923907. doi: 10.3389/fonc.2026.1923907 (PMC13547000; doi:10.3389/fonc.2026.1923907)
Supplement: Supplementary file 4 [file Table2.docx]

**Supplementary Table S2. Age- and sex-stratified diagnostic performance of DL+QEF in the temporal test cohort**

| **Subgroup** | **Patients, n** | **HB/FNH, n** | **AUC (95% CI)** | **Accuracy, % (95% CI)** | **HB sensitivity, % (95% CI)** | **FNH specificity, % (95% CI)** | **TP/FP/TN/FN** |
| --- | --- | --- | --- | --- | --- | --- | --- |
| Age <5 years | 76 | 66/10 | 0.992 (0.973–1.000) | 96.1 (89.0–98.6) | 95.5 (87.5–98.4) | 100.0 (72.2–100.0) | 63/0/10/3 |
| Age ≥5 years | 45 | 13/32 | 0.971 (0.918–1.000) | 91.1 (79.3–96.5) | 92.3 (66.7–98.6) | 90.6 (75.8–96.8) | 12/3/29/1 |
| Male | 61 | 46/15 | 0.999 (0.991–1.000) | 98.4 (91.3–99.7) | 97.8 (88.7–99.6) | 100.0 (79.6–100.0) | 45/0/15/1 |
| Female | 60 | 33/27 | 0.965 (0.919–0.996) | 90.0 (79.9–95.3) | 90.9 (76.4–96.9) | 88.9 (71.9–96.1) | 30/3/24/3 |

Note. Diagnostic performance was evaluated using the fixed classification threshold of 0.500. The 95% confidence intervals for AUCs were estimated using 5,000 stratified bootstrap resamples, and confidence intervals for accuracy, sensitivity, and specificity were calculated using the Wilson method. Subgroup analyses were exploratory, and no formal between-subgroup comparisons were performed. DL, deep learning model; QEF, quantitative enhancement feature model; HB, hepatoblastoma; FNH, focal nodular hyperplasia; AUC, area under the receiver operating characteristic curve; CI, confidence interval; TP, true positive; FP, false positive; TN, true negative; FN, false negative.
