# Supplementary material for: A pretreatment multiphasic CT-based decision-support model for differentiating pediatric hepatoblastoma from focal nodular hyperplasia
Source: Front Oncol. 2026 Aug 24;16:1923907. doi: 10.3389/fonc.2026.1923907 (PMC13547000; doi:10.3389/fonc.2026.1923907)
Supplement: Supplementary file 5 [file Table3.docx]

**Supplementary Table S3. Association of CT scanner type with diagnosis and scanner-stratified performance of the DL+QEF model**

**Panel A. Association between CT scanner type and diagnosis**

| **Cohort** | **CT scanner** | **HB, n (%)** | **FNH, n (%)** | **Total, n** | ***p* value** | **Cramér’s V** |
| --- | --- | --- | --- | --- | --- | --- |
| Training cohort | Discovery CT750 HD | 199 (92.6) | 16 (7.4) | 215 | 0.157 | 0.064 |
|  | Revolution CT | 245 (88.8) | 31 (11.2) | 276 |  |  |
| Temporal test cohort | Discovery CT750 HD | 14 (56.0) | 11 (44.0) | 25 | 0.273 | 0.100 |
|  | Revolution CT | 65 (67.7) | 31 (32.3) | 96 |  |  |

**Panel B. Scanner-stratified diagnostic performance of DL+QEF in the temporal test cohort**

| **CT scanner** | **Patients, n** | **HB/FNH, n** | **AUC (95% CI)** | **Accuracy, % (95% CI)** | **HB sensitivity, % (95% CI)** | **FNH specificity, % (95% CI)** |
| --- | --- | --- | --- | --- | --- | --- |
| Discovery CT750 HD | 25 | 14/11 | 0.981 (0.922–1.000) | 92.0 (75.0–97.8) | 92.9 (68.5–98.7) | 90.9 (62.3–98.4) |
| Revolution CT | 96 | 65/31 | 0.980 (0.951–0.999) | 94.8 (88.4–97.8) | 95.4 (87.3–98.4) | 93.5 (79.3–98.2) |

Note. In Panel A, percentages were calculated within each scanner group, and Pearson’s chi-square test was used to assess the association between CT scanner type and diagnosis within each cohort. Cramér’s V was used to quantify the strength of association. In Panel B, diagnostic performance was evaluated in the temporal test cohort using the prespecified classification threshold of 0.500. The 95% confidence intervals for AUCs were estimated using 5,000 stratified bootstrap resamples. Scanner-stratified analyses were exploratory. Wilson score intervals were calculated for accuracy, HB sensitivity, and FNH specificity. DL, deep learning model; QEF, quantitative enhancement feature model; HB, hepatoblastoma; FNH, focal nodular hyperplasia; AUC, area under the receiver operating characteristic curve; CI, confidence interval.
