# Supplementary material for: A pretreatment multiphasic CT-based decision-support model for differentiating pediatric hepatoblastoma from focal nodular hyperplasia
Source: Front Oncol. 2026 Aug 24;16:1923907. doi: 10.3389/fonc.2026.1923907 (PMC13547000; doi:10.3389/fonc.2026.1923907)
Supplement: Supplementary file 6 [file Table4.docx]

**Supplementary Table S4. Threshold sensitivity analysis using model-specific cutoffs selected from training-cohort out-of-fold predictions.**

| **Model** | **AUC (95% CI)** | **OOF Youden cutoff** | **Accuracy, % (95% CI)** | **HB sensitivity, % (95% CI)** | **FNH specificity, % (95% CI)** | **Balanced accuracy, % (95% CI)** | **PPV, % (95% CI)** | **NPV, % (95% CI)** | ***p* value vs. DL+QEF** |
| --- | --- | --- | --- | --- | --- | --- | --- | --- | --- |
| DL-PCP | 0.867 (0.803–0.925) | 0.168 | 74.4 (65.9–81.3) | 97.5 (91.2–99.3) | 31.0 (19.1–46.0) | 64.2 (57.1–71.4) | 72.6 (63.5–80.2) | 86.7 (62.1–96.3) | <0.001 |
| DL-AP | 0.900 (0.831–0.961) | 0.288 | 84.3 (76.8–89.7) | 96.2 (89.4–98.7) | 61.9 (46.8–75.0) | 79.1 (71.3–86.3) | 82.6 (73.6–89.0) | 89.7 (73.6–96.4) | 0.013 |
| DL-PVP | 0.932 (0.883–0.971) | 0.797 | 89.3 (82.5–93.6) | 87.3 (78.2–93.0) | 92.9 (81.0–97.5) | 90.1 (84.6–94.9) | 95.8 (88.5–98.6) | 79.6 (66.4–88.5) | 0.388 |
| DL-3Phase | 0.936 (0.891–0.973) | 0.450 | 86.8 (79.6–91.7) | 94.9 (87.7–98.0) | 71.4 (56.4–82.8) | 83.2 (75.5–90.3) | 86.2 (77.4–91.9) | 88.2 (73.4–95.3) | 0.039 |
| QEF | 0.955 (0.914–0.988) | 0.397 | 89.3 (82.5–93.6) | 87.3 (78.2–93.0) | 92.9 (81.0–97.5) | 90.1 (84.5–95.0) | 95.8 (88.5–98.6) | 79.6 (66.4–88.5) | 0.424 |
| DL+QEF | 0.980 (0.956–0.996) | 0.455 | 92.6 (86.5–96.0) | 97.5 (91.2–99.3) | 83.3 (69.4–91.7) | 90.4 (84.4–95.8) | 91.7 (83.8–95.9) | 94.6 (82.3–98.5) | Reference |

Note. Model-specific cutoffs were selected by maximizing the Youden index using training-cohort out-of-fold predictions and were applied unchanged to the temporal test cohort. AUCs are threshold-independent and are therefore identical to those reported in the primary analysis. HB was defined as the positive class and FNH as the negative class. Wilson score intervals were calculated for accuracy, sensitivity, specificity, PPV, and NPV; 95% confidence intervals for balanced accuracy were estimated using 5,000 bootstrap resamples stratified by diagnosis. The *p* values were calculated using exact McNemar tests comparing each model with DL+QEF at the corresponding model-specific cutoffs. DL, deep learning; QEF, quantitative enhancement feature model; OOF, out-of-fold; AUC, area under the receiver operating characteristic curve; PPV, positive predictive value; NPV, negative predictive value.
